# A Conserved Rubredoxin Is Necessary for Photosystem II Accumulation in Diverse Oxygenic Photoautotrophs*[Fn FN2]

**DOI:** 10.1074/jbc.M113.487629

**Published:** 2013-07-30

**Authors:** Robert H. Calderon, José G. García-Cerdán, Alizée Malnoë, Ron Cook, James J. Russell, Cynthia Gaw, Rachel M. Dent, Catherine de Vitry, Krishna K. Niyogi

**Affiliations:** From the ‡ Howard Hughes Medical Institute, Department of Plant and Microbial Biology, University of California, Berkeley, California 94720,; the § Physical Biosciences Division, Lawrence Berkeley National Laboratory, Berkeley, California 94720, and; the ¶ Institut de Biologie Physico-Chimique, Unité Mixte de Recherche 7141, Centre National de la Recherche Scientifique, Université Paris 6, 75005 Paris, France

**Keywords:** Arabidopsis, Chlamydomonas, Chloroplast, Electron transport, Iron-sulfur protein, Photosynthesis, Photosystem II, Synechocystis, Rubredoxin

## Abstract

In oxygenic photosynthesis, two photosystems work in tandem to harvest light energy and generate NADPH and ATP. Photosystem II (PSII), the protein-pigment complex that uses light energy to catalyze the splitting of water, is assembled from its component parts in a tightly regulated process that requires a number of assembly factors. The *2pac* mutant of the unicellular green alga *Chlamydomonas reinhardtii* was isolated and found to have no detectable PSII activity, whereas other components of the photosynthetic electron transport chain, including photosystem I, were still functional. PSII activity was fully restored by complementation with the *RBD1* gene, which encodes a small iron-sulfur protein known as a rubredoxin. Phylogenetic evidence supports the hypothesis that this rubredoxin and its orthologs are unique to oxygenic phototrophs and distinct from rubredoxins in Archaea and bacteria (excluding cyanobacteria). Knockouts of the rubredoxin orthologs in the cyanobacterium *Synechocystis* sp. PCC 6803 and the plant *Arabidopsis thaliana* were also found to be specifically affected in PSII accumulation. Taken together, our data suggest that this rubredoxin is necessary for normal PSII activity in a diverse set of organisms that perform oxygenic photosynthesis.

## Teaser

**Background:** Photosystem II is an essential component of oxygenic photosynthesis.

**Results:** Photosystem II is specifically decreased in rubredoxin mutants of the green alga *Chlamydomonas reinhardtii*, the cyanobacterium *Synechocystis* sp. PCC 6803, and the plant *Arabidopsis thaliana*.

**Conclusion:** Rubredoxin is required for photosystem II, and not photosystem I, accumulation in these organisms.

**Significance:** Rubredoxin was likely important in the evolution of oxygenic photosynthesis.

## Introduction

Photosystem II (PSII)^3^ uses absorbed light energy to catalyze the splitting of water into molecular oxygen and protons and is the only enzyme complex known to be capable of this energetically unfavorable process. Working together with photosystem I (PSI), the cytochrome *b*_6_*f* complex and the ATP synthase, PSII drives oxygenic photosynthesis and is responsible for producing most, if not all, of the oxygen present in the Earth's atmosphere (1, 2). Recent work has further refined our knowledge of the structure (3) and biogenesis (4–11) of PSII, but a complete understanding of how the 20–30 protein subunits and ∼70 cofactors are correctly assembled into a functional light-driven water:plastoquinone oxidoreductase remains elusive (12–14).

The structural and functional homology between PSII and the pheophytin-quinone (type II) reaction center of anoxygenic photosynthetic bacteria, along with many geological and biological lines of evidence, indicates that these two complexes share a common evolutionary origin and that this ancestral complex was almost certainly anoxygenic (15). Despite the availability of crystal structures for both reaction centers (3, 16), knowledge of their common ancestor is remarkably limited and therefore the evolutionary inventions and innovations that enabled a proto-PSII to use water as an electron donor (and to concomitantly release molecular oxygen) are poorly understood.

Rubredoxins are [1Fe-0S] proteins in which one iron atom is coordinated by four cysteine residues (17, 18). Most studied rubredoxins exist as small soluble proteins that act as electron carriers in a variety of biochemical processes including carbon fixation (19), detoxification of reactive oxygen species (20–22), and fatty acid metabolism (23, 24). The distribution of rubredoxins within the tree of life is distinct in that these proteins appear to be limited to divergent groups within the archaea and bacteria, whereas they are found in only one group of eukaryotes: plants and photosynthetic algae that contain a plastid derived ultimately from primary endosymbiosis of a cyanobacterium. Interestingly, this eukaryotic “photosynthetic” rubredoxin is thylakoid membrane-associated and, together with the homologous protein found in cyanobacteria, appears to represent a class of rubredoxins unique to the oxygenic photoautotrophs that is distinct from all other known rubredoxins (25, 26).

Previous studies of thylakoid membrane rubredoxins have yielded conflicting results. Analysis of a mutant strain of the cyanobacterium *Synechococcus* sp. PCC 7002 lacking *rubA*, the membrane-bound rubredoxin, led to the conclusion that the function of this protein was to aid in iron-sulfur cluster assembly of PSI and that it had little or no effect on PSII (26, 27). Surprisingly, however, an antibody raised against the membrane-bound rubredoxin from the photosynthetic cryptophyte *Guillardia theta* was found to react with a homologous protein in PSII-enriched particles from spinach (28).

Here we report the characterization of mutants lacking the thylakoid membrane-associated rubredoxin in the green alga *Chlamydomonas reinhardtii,* the cyanobacterium *Synechocystis* sp. PCC 6803 and the flowering plant *Arabidopsis thaliana*. We show that these mutants exhibit a PSII-specific defect and that the role of this rubredoxin in contributing to the assembly or stability of PSII is likely conserved in oxygenic photoautotrophs.

## EXPERIMENTAL PROCEDURES

### 

#### 

##### Strains, Mutant Generation, and Growth Conditions

*Chlamydomonas reinhardtii* wild-type strain 4A+ and the mutant strains *2pac*, gRBD1–1, gRBD1–2, and Fud7 (29) were maintained on Tris acetate-phosphate (TAP) medium (30) in the dark and at 25 °C unless otherwise indicated. The *2pac* mutant (CAL028.03.28) was generated by insertional mutagenesis of the 4A+ strain with linearized pBC1 plasmid encoding paromomycin resistance (31), and flanking sequence was isolated by SiteFinding PCR (32) using the primers listed in supplemental Table S1. SiteFinding PCR products were separated by gel electrophoresis and isolated using the QIAquick gel extraction kit (Qiagen) before sequencing. Generation of gRBD1 complemented lines was achieved by transformation of the *2pac* mutant with a 1.75 kb fragment of genomic DNA (for primers see supplemental Table S1) that includes the 510 bp coding sequence of the *RBD1* gene cloned into the Gateway® vector pENTR-D (Invitrogen), as described (33). After transformation, cells were allowed to recover in 10 ml of TAP overnight in the dark with shaking at 110 rpm. Cells were then collected by centrifugation (1,300 × *g*, 3 min), resuspended in 300 μl of high-salt (HS) medium (34), and plated onto HS agar plates. The plates were maintained under 50 μmol of photons m^−2^ s^−1^ for 3 to 4 weeks before the autotrophic transformed colonies were picked.

The glucose-tolerant strain of the cyanobacterium *Synechocystis* sp. PCC 6803 (35) was used as a parental strain for generation of the Δ*rubA* (Δ*slr2033*) mutant and as a wild-type control. All strains were grown on solid or in liquid BG-11 medium (36) buffered with 25 mm Hepes-NaOH, pH 7.5 and supplemented with 5 mm glucose at 32 °C under constant illumination (30 μmol of photons m^−2^ s^−1^) unless otherwise indicated.

The Δ*rubA* mutant was obtained via transformation of wild-type cells as described (36) with a construct consisting of the 5′ and 3′ flanking regions of the *rubA* (*slr2033*) coding sequence directly fused with a cassette containing *nptI* (encoding resistance to kanamycin) and *sacB* (conferring lethality in the presence of sucrose) (37). Transformants were selected on BG-11 plates supplemented with 5 mm glucose, 5 μm DCMU, and 50 μm kanamycin. Segregation was analyzed by PCR amplification of both the flanking region and the coding sequence of *rubA* (for primers, see supplemental Table S1). Complementation of the Δ*rubA* mutant was achieved by transformation with a plasmid containing the 5′ and 3′ flanking regions of the *slr0168* coding sequence surrounding a construct consisting of the *psbA2* promoter (38) fused with the coding sequence of *rubA*, allowing for incorporation into a neutral site as described (39).

Seeds from *Arabidopsis thaliana* wild type (Col-0) and from mutant GT12976 obtained from the Cold Spring Harbor GeneTrap collection (40, 41) were sterilized and plated on plant nutrient plates (42) supplemented with 0.5% sucrose (5g/liter) (and kanamycin (50 μg/ml), where indicated). Primers used for genotyping are listed in supplemental Table S1.

##### Chlorophyll Fluorescence

Minimum (F_o_), maximum (F_m_), and variable (F_v_/F_m_) chlorophyll fluorescence of *Chlamydomonas, Synechocystis,* and *Arabidopsis* lines were measured after 15 min of dark acclimation with a pulse-amplitude-modulated fluorescence imaging system (MAXI-IMAGING-PAM, Heinz Walz, Effeltrich, Germany).

Fluoresence induction kinetics were monitored before and after addition of 10 μm DCMU on custom-built equipment as described (43).

##### Protein Analysis

For all samples, chlorophyll concentration was determined as described (44). For isolation of total protein from *Chlamydomonas*, cells were pelleted and washed in wash buffer (5 mm Hepes-NaOH, pH 7.5, 10 mm EDTA, 1 mm benzamidine-HCl, 5 mm aminocaproic acid and 200 μm PMSF). After washing, cells were pelleted and resuspended in resuspension buffer (200 mm DTT, 200 mm Na_2_CO_3_, 1 mm benzamidine-HCl, 5 mm aminocaproic acid, and 200 μm PMSF) and solubilized by further addition of 5% SDS/30% sucrose solution to final concentration of 2% SDS/12% sucrose. Samples were mixed, boiled for 50 s, placed on ice and centrifuged. For isolation of total protein from *Synechocystis,* cells were harvested and incubated with lysozyme (0.05% *w*/*v*) at 37 °C for 45 min before sonicating to break open cells. Supernatant was isolated and solubilized in 2× Laemmli buffer (45) for 30 min at 55 °C. *Arabidopsis* protein extraction was performed as described (46). 2 μg (*Chlamydomonas*) or 0.5 μg (*Synechocystis*) of chlorophyll were loaded per “100%” lane while loading based on equal amounts of AtpB was performed for each “100%” *Arabidopsis* lane. Samples were run on 10–20% Tris-Glycine gels (Invitrogen) followed by semi-dry transfer to a PVDF membrane. Specific polyclonal antibodies were used and signals were visualized by Supersignal West Femto Chemiluminescent substrate detection system (Thermo Scientific). Polyclonal antibodies against AtpB, D2, RbcL, and CP43 were obtained from Agrisera (Sweden). The antibodies against D1, PsaA/PsaD, cytochrome *f*, PsaC, and RubA were kind gifts from Profs. A. Melis, J.-D. Rochaix, R. Malkin, A. Haldrup, and D. Bryant, respectively. For the production of an antibody against the RBD1 and AtRBD1 proteins (excluding the transmembrane helix), portions of the *RBD1* and *AtRBD1* cDNA were subcloned (using the primers listed in supplemental Table S1) into the pET28 (a+) vector (Novagen). These constructs were introduced into *Escherichia coli* strain BL21 (DE3) (Novagen), and protein expression was induced by addition of IPTG. The His-tagged RBD1 and AtRBD1 proteins were purified under native conditions through a Ni-NTA column (Qiagen) according to the manufacturer's instructions. Specific polyclonal antibodies were generated in rabbits by ProSci Inc.

##### Absorption Spectroscopy

Electrochromic band shift and redox changes of P700 were assessed by monitoring absorbance at 520 and 705 nm, respectively, with a JTS-10 spectrophotometer (BioLogic) as previously described (43, 47, 48). *Chlamydomonas* and *Synechocystis* cells were dark-acclimated for 10 min in 20 mm Hepes, 20% Ficoll, pH 7.2. A 5 ns and 1 mJ cm^−2^ laser flash was used to activate light reactions for electrogenicity measurements. To achieve mildly reducing conditions, cells were thoroughly mixed and aerated before absorbance measurement for each wavelength probed. PSII was inhibited by addition of 1 mm HA and 10 μm DCMU. P700 measurements of *Arabidopsis* were performed on a one leaf-thick array of leaves after 10 min of dark acclimation.

##### Phylogenetic Tree Construction

The indicated sequences were retrieved from UniProt and aligned using PRANK (49). Putative transit peptides and poorly aligning regions were manually removed and the remaining sequences were realigned (supplemental Dataset S1). PhyML 3.0 (50) was used to construct a maximum likelihood tree from the alignment file and an SH-like approximate likelihood ratio test (51) was performed to calculate support for each branch. Support for the oxygenic photoautotroph branch was consistently over 0.85 and was not substantially changed by the removal of any one sequence.

## RESULTS

### 

#### 

##### The 2pac Mutant Exhibits No Detectable PSII Activity

**FIGURE 1.**
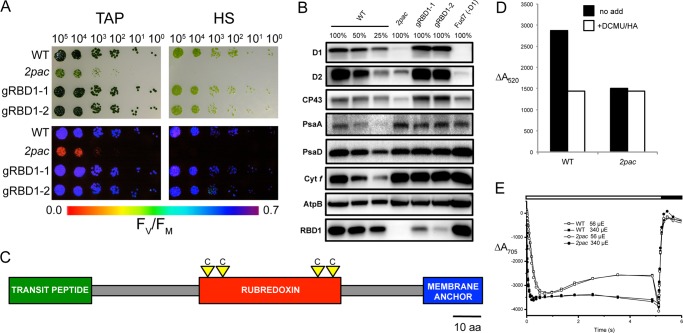
**Growth and phenotypic comparisons of wild-type (4A+), *2pac*, two complemented lines (gRBD1–1 and gRBD1–2) and a strain lacking the *psbA* gene encoding the D1 protein (Fud7).** *A*, growth (*upper panels*) and variable fluorescence (F_v_/F_m_, *lower panels*) of strains grown on plates with (TAP) and without (HS) acetate at 30 μmol photons m^−2^ s^−1^. *B*, immunoblot analysis of steady-state levels of PSII (D1, D2, and CP43), PSI (PsaA and PsaD), cytochrome *b*_6_*f* (Cyt *f*), and ATP synthase (AtpB) subunits, as well as of RBD1. *C*, model of the RBD1 protein showing predicted N-terminal chloroplast transit peptide (*green*), rubredoxin domain (*red*, with locations of conserved iron-coordinating cysteines denoted by *yellow triangles*) and C-terminal transmembrane helix (*blue*). *D*, phase *a* component of *A*_520 nm_, a measurement of the electrochemical gradient formed after illumination, before (*black*) and after (*white*) treatment with the PSII-specific inhibitors DCMU and HA. *E*, P700 oxidation and reduction kinetics of wild type (WT) and *2pac,* measured by absorbance at 705 nm after treatment with DCMU and HA.

A collection of acetate-requiring *Chlamydomonas* insertional mutants (52) was grown in the dark and screened for mutants displaying a very low maximum efficiency of PSII (F_v_/F_m_). One such mutant, CAL028.03.28, was renamed *second photosystem assembly component* (*2pac*) and selected for further characterization. The *2pac* mutant was unable to grow photoautotrophically on minimal medium and grew more slowly than the wild-type strain when grown mixotrophically (Fig. 1*A*). Analysis of chlorophyll *a* fluorescence revealed an initial fluorescence (F_o_) level that was equivalent to the maximum fluorescence (F_m_), resulting in a lack of variable fluorescence (F_v_) from PSII and an F_v_/F_m_ value of 0 (Fig. 1*A*).

Steady-state levels of proteins from the photosynthetic electron transport chain were assayed by immunoblot analysis, and a ∼90% decrease in the levels of the PSII reaction center proteins D1, D2, and CP43 in the *2pac* mutant was observed (Fig. 1*B*). These immunoblot data, along with the growth and chlorophyll fluorescence results, indicate that the *2pac* insertional mutant is unable to perform photosynthesis and that this is likely due to the absence of PSII activity.

##### The PSII-deficient Phenotype of 2pac Is Due to the Absence of the RBD1 Gene

**FIGURE 2.**
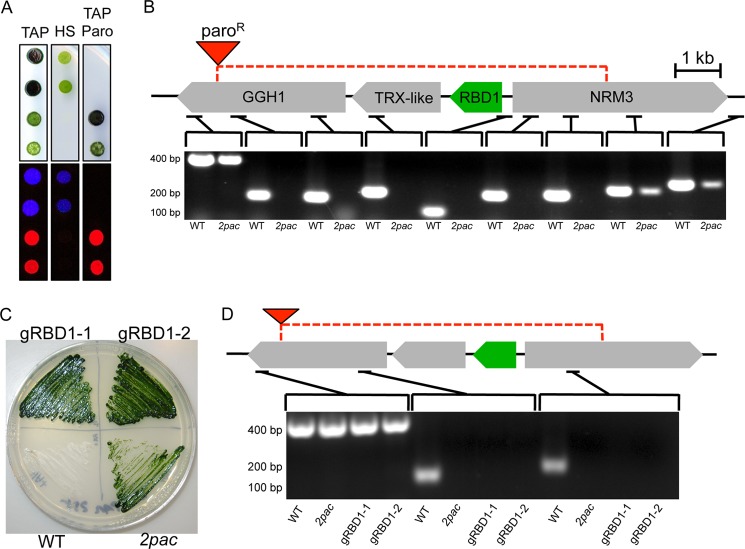
**The absence of the *RBD1* gene is responsible for the loss of variable fluorescence.** *A*, representative tetrad obtained from the backcross of *2pac* with 4A- (wild-type) indicates that the insertion cosegregates with the PSII-deficient phenotype. A total of 45 progeny were obtained (some from incomplete tetrads), and 19 were found to retain resistance to paromomycin and have no variable fluorescence. The remaining 26 were susceptible to paromomycin and displayed wild-type levels of variable fluorescence. *B*, PCR amplification of small fragments of genomic DNA showed that the *2pac* mutant contains a ∼12.5 kb deletion spanning four genes. *C*, two independent lines generated by transformation of *2pac* with a construct containing *RBD1* (gRBD1–1 and gRBD1–2) retained resistance to paromomycin. *D*, PCR analysis showed that gRBD1–1 and gRBD1–2 also have the deletion present in *2pac*.

Tetrad analysis of a backcross of the *2pac* mutant to the wild-type strain showed that the mutant phenotypes (acetate requirement and absence of variable fluorescence) segregated together in a 2:2 pattern, indicating that they are due to a single nuclear mutation (Fig. 2*A*). Both phenotypes were linked to the paromomycin resistance that was used as the selectable marker for insertional mutagenesis (Fig. 2*A*), suggesting that the *2pac* mutation was tagged by the insertion of the antibiotic resistance gene.

SiteFinding PCR (32) was used to identify the location of the insertion in the *2pac* mutant. Sequence flanking the insertion was recovered that corresponded to a region on chromosome 7 within a gene encoding a putative γ-glutamyl hydrolase (Cre07.g315050). Amplification of small (≤400 bp) segments of genomic DNA upstream and downstream of the insertion revealed an adjacent deletion of ∼12.5 kb affecting three additional genes: Cre07.g315100, Cre07.g315150/*RBD1*, and Cre07.g315200/*NRM3* (Fig. 2*B*). BLAST searches revealed that only one of these genes, *RBD1*, has homologs in all oxygenic photoautotrophs. This gene encodes a 169 amino acid protein that includes a predicted N-terminal chloroplast transit peptide, a rubredoxin domain and a predicted C-terminal transmembrane helix (Fig. 1*C*). Using a specific antibody raised against recombinant RBD1, the RBD1 protein was detected in the wild type but not in the *2pac* mutant (Fig. 1*B*).

To test whether or not the absence of RBD1 was responsible for the PSII-deficient phenotype of *2pac*, a 1.75-kb fragment of genomic DNA containing the *RBD1* gene was amplified from the wild type. This fragment, which included ∼1 kb of sequence upstream of the predicted start codon, the coding sequence and ∼200 bp downstream of the predicted stop codon, was used to transform the *2pac* mutant. Two strains independently generated by this transformation, denoted gRBD1–1 and gRBD1–2, showed wild-type levels of variable fluorescence and photoautotrophic growth (Fig. 1*A*), as well as restored levels of D1, D2, CP43 and the RBD1 protein (Fig. 1*B*). Both complemented lines also retained resistance to paromomycin and the 12.5 kb deletion, indicating that the restoration of photoautotrophic growth and variable fluorescence was due specifically to complementation of the *2pac* mutation by the *RBD1* gene (Fig. 2, *C* and *D*).

##### PSII, but Not Other Major Photosynthetic Electron Transport Complexes, Is Defective in 2pac

Because previous work had shown that the homolog of RBD1 (RubA) was essential for PSI assembly in the cyanobacterium *Synechococcus* sp. PCC 7002 (26, 27), we assayed the activity of other complexes functioning in the photosynthetic electron transport chain. Photosynthetic electron transport results in a trans-thylakoid electric field that alters the absorbance spectra of pigments within the membrane, a phenomenon known as electrochromism (53). To determine the relative contributions of PSII and PSI to the light-induced electrochemical gradient across the thylakoid membrane, we measured these changes in absorbance at 520 nm (Δ*A*_520 nm_) upon excitation with a saturating laser pulse. Addition of PSII-specific inhibitors DCMU and HA to wild-type cells eliminated the contribution of PSII to the Δ*A*_520 nm_, with the remaining activity attributable to PSI (Fig. 1*D*). The *2pac* mutant showed only a contribution of PSI to Δ*A*_520 nm_, since it was not affected by addition of DCMU and HA (Fig. 1*D*), demonstrating that PSII does not contribute to the electrochemical gradient in *2pac*. To further assay PSI activity, light-induced absorbance changes at 705 nm (P700) were measured at both 56 and 340 μmol photons m^−2^ s^−1^ in the presence of DCMU and HA. In both cases, P700 absorbance and oxidation/reduction kinetics in *2pac* were indistinguishable from that of wild-type (Fig. 1*E*). Altogether, these spectroscopic data are consistent with the specific absence of a functional PSII in *2pac*.

##### RBD1 and Its Homologs Have a Unique Domain Architecture and Are Present in All Sequenced PSII-containing Organisms

**FIGURE 3.**
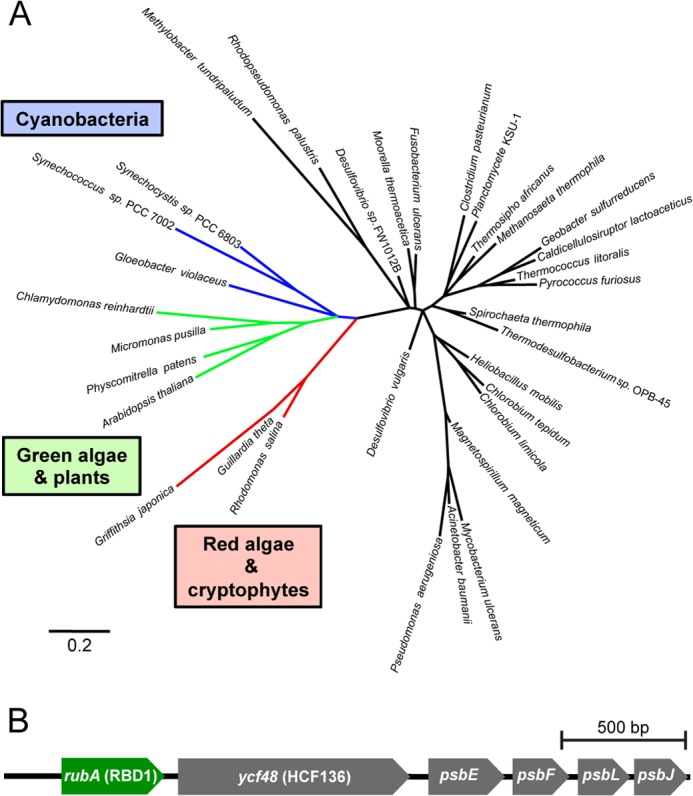
**Phylogenomic and phylogenetic analyses support a conserved role for the membrane-bound rubredoxin in PSII accumulation.** *A*, phylogenetic reconstruction of the relationship of rubredoxins from indicated species. *B*, highly-conserved six gene arrangement encoding *rubA* (RBD1), a PSII assembly factor (*ycf48*), and four PSII subunits is found in nearly all sequenced cyanobacteria, with exceptions noted under “Results.”

Genes encoding proteins with high identity to RBD1 were found in all sequenced organisms known to perform oxygenic photosynthesis. RBD1 and its orthologs in oxygenic phototrophs have a rubredoxin domain fused to a C-terminal transmembrane helix (25). This helix likely anchors RBD1 in the thylakoid membrane as evidenced by detection of the RBD1 homolog in highly-purified thylakoid membranes from *Synechococcus sp*. PCC 7002 (26) and in the thylakoid membrane proteome of *Arabidopsis thaliana* (54). The canonical rubredoxin from other bacteria and archaea, however, is usually found as a soluble protein consisting almost entirely of the rubredoxin domain, although a number of other forms of rubredoxin domain-containing proteins are also found, most notably rubrerythrins (55). A phylogenetic reconstruction of the relationship between representative rubredoxins from different species shows that those from PSII-containing organisms (RBD1 homologs) form a clade distinct from all others (Fig. 3*A*), suggesting that a membrane-bound rubredoxin was almost certainly present in the most recent common ancestor of all oxygenic cyanobacteria and plastids.

All sequenced cyanobacteria, with several interesting exceptions, contain a conserved six-gene arrangement that includes *RBD1* (called *rubA* in cyanobacteria), a known (7, 9) PSII assembly factor (*HCF136*/*ycf48*) and four subunits of PSII (*psbEFJL*) (Fig. 3*B*). In three thermophilic cyanobacterial isolates *Thermosynechococcus elongatus BP-1*, *Synechococcus* sp. JA-2–3B'a (2–13), and *Synechococcus* sp. JA-3–3Ab, this six gene arrangement has been separated into an *RBD1*/*rubA-HCF136*/*ycf48* cluster and a *psbEFLJ* cluster. In one isolate (strain MBIC11017) of the chlorophyll *d*-containing cyanobacterium *Acaryochloris marina,* this arrangement appears to have been duplicated with each duplicate undergoing gene loss, but still maintaining at least one copy of each of the six genes. Lastly, in the recently identified and sequenced UCYN-A (56), a nitrogen-fixing cyanobacterial isolate that lacks PSII (but retains PSI) and therefore is incapable of oxygenic photosynthesis, there appears to be no *RBD1* homolog present in the genome.

##### RBD1 Knockouts in Synechocystis sp. PCC 6803 and Arabidopsis thaliana Also Display a PSII-specific Phenotype

**FIGURE 4.**
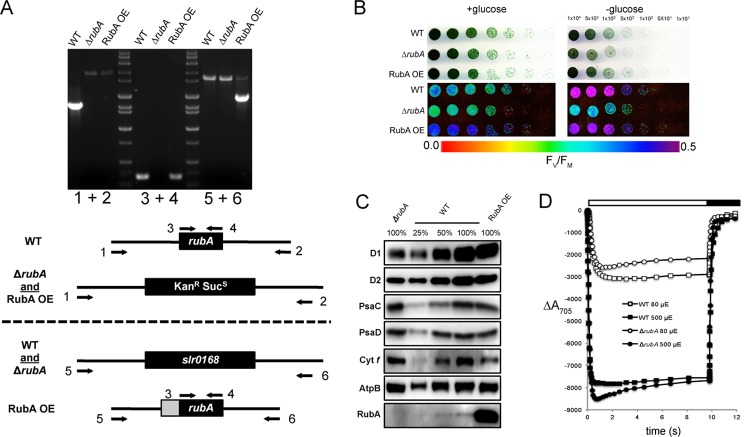
**Characterization of the Δ*rubA* mutant of the cyanobacterium *Synechocystis sp.* PCC 6803.** *A*, genotyping of the mutant strains was performed via PCR amplification of both the flanking region of *rubA* (using primers 1 and 2), the *rubA* coding sequence (CDS, using primers 3 and 4) and neutral site used for RubA overexpression (*slr0168*, using primers 5 and 6) from the wild type (WT), Δ*rubA* mutant and RubA OE strain. *B*, growth (*upper panels*) and variable fluorescence (F_v_/F_m_, *lower panels*) of wild type (*WT*), Δ*rubA* and a complemented line (RubA OE) grown on plates with and without glucose. *C*, immunoblot analysis of WT, Δ*rubA*, and RubA OE. *D*, P700 oxidation and reduction kinetics measured on equal numbers of cells of WT and Δ*rubA* mutant.

The phylogenetic analysis of rubredoxins and the phenotype of the *2pac* mutant led us to hypothesize that *RBD1*, and its homologs might have a conserved function in all oxygenic phototrophs. To test this hypothesis, we analyzed *rbd1* mutants in a model cyanobacterium (*Synechocystis* sp. PCC 6803) and plant (*A. thaliana*). First, we produced a Δ*rubA* mutant strain of the glucose-tolerant cyanobacterium *Synechocystis* sp. PCC 6803 in which the gene *rubA*/*RBD1*(*slr2033*) was replaced with a cassette encoding resistance to the antibiotic kanamycin (*nptI*) and sensitivity to sucrose (*sacB*). After multiple rounds of restreaking on kanamycin plates, full segregation for the deletion was achieved and confirmed via PCR (Fig. 4*A*). The Δ*rubA* strain was able to grow photoautotrophically but showed reduced variable fluorescence relative to wild-type (∼70%) under both mixotrophic and photoautotrophic growth (Fig. 4*B*), suggesting a defect in PSII. Immunoblot analysis of wild-type and Δ*rubA* strains revealed that the Δ*rubA* strain has highly reduced levels of D1 and D2 (∼ 40%), lacks the RubA protein, but retains wild-type levels of cytochrome *f* and PSI subunits PsaC and PsaD (Fig. 4*C*). To directly assess the functionality of PSI, we measured changes of absorbance at 705 nm (P700) at 80 and 500 μmol of photons m^−2^ s^−1^ in the presence of DCMU on equal cell densities of wild-type and Δ*rubA*. Both strains showed P700 redox changes of similar amplitude and kinetics (Fig. 4*D*), pointing to the presence of wild-type levels of functional PSI in Δ*rubA* cells. To ensure the PSII defect was due specifically to the loss of RubA, the Δ*rubA* mutant was transformed with a construct that allowed for overexpression of *rubA* in a neutral site in the genome, generating the RubA OE strain. The RubA OE strain displayed variable fluorescence that was restored to approximately wild-type levels (Fig. 4*B*). Immunoblot analysis of the RubA OE strain showed that the RubA protein was present at much higher levels than in the wild-type and that PSII subunits D1 and D2 were restored to wild-type levels (Fig. 4*C*). While overexpression of RubA appears to have caused a reduction in levels of cytochrome *f* and PSI subunits, it did not affect photosynthetic electron transport downstream of PSII, as fluorescence induction kinetics were indistinguishable between the wild-type and RubA OE strains (data not shown).

**FIGURE 5.**
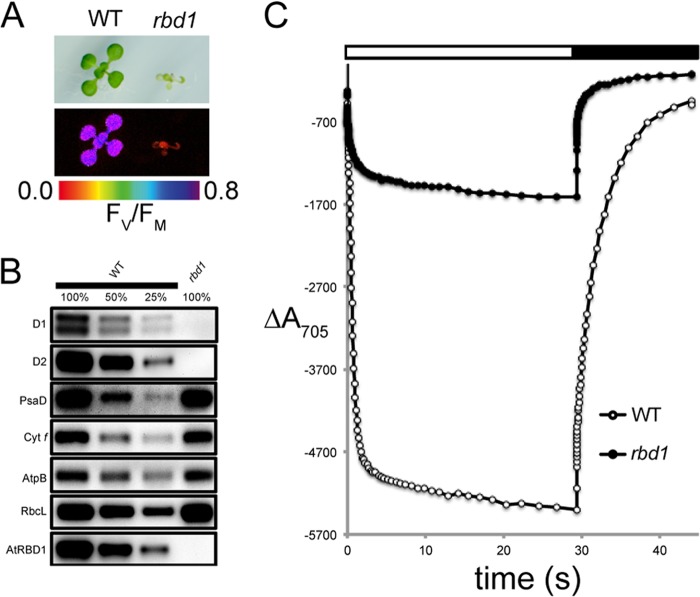
**Characterization of *A. thaliana* T-DNA lines homozygous for an insertion within *At1g54500,* the *RBD1* ortholog.** *A*, growth (*upper panels*) and variable fluorescence (F_v_/F_m_, *lower panels*) of wild type (*WT*) and a plant homozygous for an insertion within the *RBD1* gene (*rbd1*). *B*, immunoblot analysis of WT and homozygous *rbd1* plants. *C*, P700 oxidation kinetics of wild-type and homozygous *rbd1* plants.

The ortholog of RBD1 in the flowering plant *A. thaliana* is encoded by the gene *At1g54500*. An *Arabidopsis* mutant heterozygous for a transposon insertion within *At1g54500* was obtained, and seeds harvested from the self-fertilized plant were grown on plates containing 0.5% sucrose to support growth of potential non-photosynthetic mutants. Seedlings were genotyped by PCR (data not shown), and those homozygous for the insertion were pale green, showed no detectable variable chlorophyll *a* fluorescence and had an F_v_/F_m_ value of 0 (Fig. 5*A*), indicating a lack of PSII activity. The F_v_/F_m_ values of homozygous wild-type progeny were ∼0.75 (Fig. 5*A*), the same value as measured for heterozygous progeny (data not shown). Immunoblot analysis showed that subunits of PSII (D1 and D2) were also severely reduced in the mutant while other components of the photosynthetic apparatus (PSI, cytochrome *b*_6_*f* and the ATP synthase) were unaffected (Fig. 5*B*). Furthermore, using an antibody raised against recombinant AtRBD1, we were able to detect the AtRBD1 protein in the wild-type but not in the mutant. We measured PSI activity by examining the kinetics of P700 oxidation and reduction and found that plants homozygous for the insertion exhibited wild-type kinetics, although with a weaker amplitude (Fig. 5*C*), similar to recent results with a mutant specifically lacking PSII (48). To ensure that the insertion and PSII phenotype were genetically linked, we grew seedlings on plates containing 0.5% sucrose and kanamycin (to select for plants harboring the insertion). Nineteen out of 58 plants (∼33%) were kanamycin sensitive and died at the cotyledon stage, which was the same phenotype exhibited by the wild-type controls (data not shown). 26 out of 58 plants (∼45%) were kanamycin resistant and displayed wild-type levels of variable fluorescence (F_v_/F_m_ ∼ 0.75). The remaining 13 plants (∼22%) grew past the cotyledon stage but were pale and lacked PSII activity, as evidenced by high initial levels of fluorescence and no detectable variable fluorescence. The observed results are consistent with a 1:2:1 Mendelian ratio (X^2^ = 1.862, *p* < 0.39), suggesting that the PSII deficiency is caused by a single, recessive mutation in *At1g54500*.

## DISCUSSION

Several lines of evidence show that the thylakoid-associated rubredoxin encoded by *RBD1* is necessary specifically for PSII activity in *Chlamydomonas*. Without RBD1, the *2pac* mutant does not accumulate PSII as assayed by chlorophyll *a* fluorescence (Fig. 1*A*), immunodetection of PSII subunits (Fig. 1*B*), and measurements of ΔA_520 nm_ (Fig. 1*D*). PSII accumulation is clearly restored via transformation of the *2pac* mutant with a small fragment of DNA containing only *RBD1*, its putative promoter and its 3′ UTR (Fig. 1). The mature RBD1 protein is small (∼16 kDa) and is a membrane-associated rubredoxin (Fig. 1*B*). Membrane-bound rubredoxins are found exclusively in PSII-containing organisms and are distinct from the well-studied soluble rubredoxins found in many archaea and bacteria (Fig. 3*A*). The PSII-specific defect of *Synechocystis* (Fig. 4) and *Arabidopsis* (Fig. 5) mutants lacking a RBD1 homolog indicates that the role of RBD1 in PSII assembly or stability is broadly conserved in a diverse group of oxygenic photoautotrophs. The residual amount of PSII in the cyanobacterial mutant compared with the complete absence in eukaryotic mutants is consistent with previously observed mutants in PSII assembly factors (4, 7, 9) and is thought to be due, at least in part, to more efficient quality control mechanisms in plastids than in their cyanobacterial relatives (4, 57). Interestingly, PSII activity as assayed by variable fluorescence is lower than in the wild-type but apparently sufficient to support photoautotrophic growth (Fig. 4*B*), providing an opportunity for future studies into the precise functional role of RBD1 in the proper biogenesis of PSII.

Our results are in agreement with previous work showing that the membrane-bound rubredoxin is present in spinach PSII preparations (28). However, our findings with *Chlamydomonas*, *Synechocystis*, and *Arabidopsis* differ from reports on a *rubA* mutant of the cyanobacterium, *Synechococcus* sp. PCC 7002, which lacked PSI but not PSII activity due to a defect in iron-sulfur cluster assembly (26, 27). This discrepancy could simply be due to a difference in RubA function in different cyanobacterial species, despite the conserved genomic location of the *rubA* gene next to five other genes involved in PSII function (Fig. 3*B*). In *Chlamydomonas* and *Arabidopsis*, there appear to be multiple chloroplast-localized rubredoxins, so it is possible that one of these other homologs functions in PSI assembly. However, *Chlamydomonas* RBD1 (and its *Arabidopsis* and *Synechocystis* homologs encoded by *At1g54500* and *rubA,* respectively) does not seem to play a major role in PSI assembly as evidenced by measurements of Δ*A*_520 nm_ (Fig. 1*D*), P700 redox changes (Figs. 1*E*, 4*D*, and 5*C*), as well as PsaA, PsaC and PsaD protein accumulation (Figs. 1*B*, 4*C*, and 5*B*).

Because PSII activity is fully restored in the complemented lines gRBD1–1 and gRBD1–2 despite relatively low levels of RBD1 protein accumulation (Fig. 1), RBD1 is unlikely to be a subunit of PSII, rather it may have a catalytic, substoichiometric role in promoting PSII assembly or stability. This is further supported by the detection of wild-type levels of RBD1 in the Fud7 mutant lacking the D1 protein (Fig. 1*B*). It is known that rubredoxins participate in electron transport reactions in some Archaea and bacteria. Indeed, the midpoint redox potential of the RBD1 homolog from the cryptophyte alga *Guillardia theta* was found to be ∼ +125 mV (25), a value that could enable it to participate in electron transport with, or perhaps bypassing, plastoquinone (E_m_ ∼ +100 mV, (1)). In fact, a similar role has been suggested for a pair of flavodiiron proteins that function in the photoprotection of PSII in *Synechocystis* (58).

Rubredoxins have also been previously described as aiding in oxygen tolerance, either by reacting with reactive oxygen species directly or by helping to maintain the appropriate redox state of Fe-containing active sites in some enzymes (20–22). PSII, which generates oxygen and contains both a redox-active non-heme Fe as well as a redox-active heme (cytochrome *b*_559_), would certainly fit the profile of a protein complex that might benefit from the presence of such an enzyme. In many microbes, the active site of superoxide reductase (SOR) contains a non-heme iron that, after catalyzing the reduction of superoxide to hydrogen peroxide, is thought to be reactivated via rubredoxin-mediated re-reduction (59, 60). Cytochrome *b*_559_ has recently been shown to have superoxide reductase and oxidase activity (61) and the presence of *RBD1/rubA* in a highly conserved gene cluster with the two subunits of this transmembrane cytochrome might also be taken to suggest an as yet unidentified interaction between these proteins.

Another possible clue regarding the function of RBD1 may be found by examination of the synthesis of the membrane-bound [NiFe] uptake hydrogenase. The gene cluster required for synthesis of this hydrogenase is well conserved among anaerobic and aerobic species, but aerobic species with this enzyme contain an additional operon in which one of the genes (*hupI* or *hoxR*) encodes a rubredoxin (20, 22). These rubredoxins appear to contribute to oxygen tolerance of the hydrogenase, and it is of note that some of these species are photosynthetic purple bacteria that contain type II reaction centers, which are believed to be similar to the ancestral state of PSII (1). Although they are not required for assembly of anoxygenic type II reaction centers in bacteria, it is tempting to speculate that these types of soluble rubredoxins might be representative of the ancestral state of the rubredoxin motif and that the membrane-bound rubredoxin found in the oxygenic phototrophs might be an evolutionary innovation co-opted from a soluble ancestral protein during the transition from anoxygenic to oxygenic photosynthesis.

## Footnotes

3 The abbreviations used are:

PSII: photosystem II
PSI: photosystem I
DCMU: 3-(3,4-dichlorophenyl)-1,1-dimethylurea
HA: hydroxylamine
TAP: Tris acetate-phosphate.
